# Transoral Penetrating Foreign Body Into the Foramen Magnum: A Case Report

**DOI:** 10.7759/cureus.72494

**Published:** 2024-10-27

**Authors:** Astrid Rosero-Castillo, Carlos Alfredo Gómez-de la Cruz, Jacinto Eduardo Treviño-Cárdenas, Adrian Barragán-Tinajero, Jose Treviño-González

**Affiliations:** 1 Otolaryngology - Head and Neck Surgery, Hospital Universitario Dr. José Eleuterio González, Universidad Autonoma De Nuevo Leon, Monterrey, MEX; 2 Otolaryngology - Head and Neck Surgery, Hospital Universitario Dr. José Eleuterio González, Monterrey, MEX

**Keywords:** foramen magnum, foreing body, pediatric trauma, skull base injury, transoral penetrating injury

## Abstract

In this paper, we present the case of a four-year-old boy with a penetrating transoral injury caused by a foreign object directed toward the foramen magnum. Head angiotomography revealed that the object's pathway was in close contact with the vertebral artery, without apparent involvement of the meninges. We discuss the clinical presentation, diagnostic approach, and treatment in this case.

## Introduction

Young children tend to run while holding objects in their hands or mouth, predisposing them to impalement injuries [1]. The most frequent objects reported include pencils, screwdrivers, sewing needles, chopsticks, knives, and scissors [2]. The clinical presentation of penetrating injuries in the craniofacial region has various pathological conditions such as bone fractures and a specific concern for vascular injuries, as well as lacerations of meninges or brain structures. Penetrating traumatic brain injury represents approximately 0.4% of all injuries affecting the head. It can occur transcranially, transorbitally, transnasally, and transorally, with the involvement of the skull base being rare [3,4]. Children under two years of age are particularly vulnerable to neurological injuries due to incomplete ossification of the skull.

It is crucial to take a timely approach through appropriate diagnostic and radiological tests [5]. To manage these cases a multidisciplinary approach is necessary to ensure uneventful healing of these lesions and restore normal function [6]. Although various traumatic penetrating injuries to the brain have been described, to our knowledge, only a few reports have involved a penetrating injury via the transoral route to the foramen magnum [5,7]. Therefore, we present an unusual transoral penetrating trauma to the foramen magnum case by a paintbrush, the history, radiographic studies, and treatment are presented, and discuss its management.

## Case presentation

A four-year-old male, with a complete immunization schedule for his age, weighing 15 kg and measuring 110cm in height, presented to the pediatric emergency department of a tertiary care hospital 12 hours after falling from a height of approximately one meter while holding a paintbrush. The distal portion of the paintbrush had penetrated through the oral cavity towards the nasopharyngeal region.

**Figure 1:**
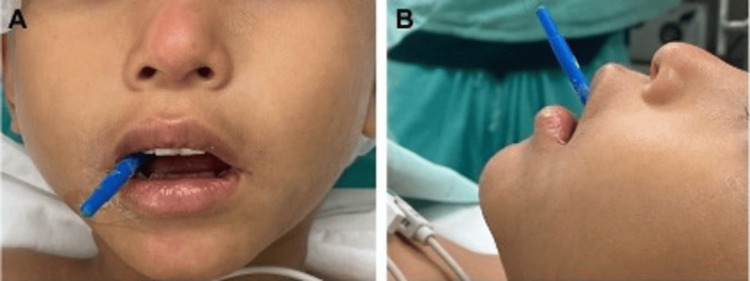
(A, B) Foreign body in oral cavity on initial evaluation.

The patient was alert and oriented with a Glasgow Coma Scale of 15/15, afebrile, and showed no signs of respiratory distress. On arrival, the initial vital signs were as follows: blood pressure of 100/60 mmHg, heart rate of 101 beats per minute, respiratory rate of 18 breaths per minute, temperature of 36.5°C, and oxygen saturation of 99%. On examination, a foreign plastic object was observed passing through the base of the uvula in a posterior and superior direction without active bleeding (Figures 1A, 1B). The patient showed no signs of head trauma or fractures. Upon admission, the emergency pediatrics department recommended fasting, continuous monitoring of vital signs, analgesia with ketorolac and acetaminophen, and antibiotic coverage with metronidazole, and cefalotin adjusted to the patient's weight. Laboratory tests were requested, along with a computed tomography angiography of the head and consultations with the neurosurgery and otorhinolaryngology departments. The laboratory tests are shown in Table 1.

**Table 1:** Laboratory and blood gas findings.

Parameter	Values	Range (uit)
Hemoglobin	15.6	12.2-18.1 (g/dL)
Leukocytes	13.2	4.00-11.00 (K/µL)
Neutrophils	10.1	2.00-6.90 (K/µL)
Lymphocytes	2.08	0.60-3.40 (K/µL)
Platelets	283	142.00-424.00 (K/µL)
Coagulation tests		
Prothrombin time	34.7	9.89 -12.6 (seconds)
Activated partial thromboplastin time	12.8	28.5 - 36.8 (seconds)
INR	1.14	
Blood gas analysis		
pH	7.45	7.32-7.43
pCO_2_	34	40-45 (mmHg)
HCO_3_	23.6	34.0-30.0 (mmol/L)

**Figure 2:**
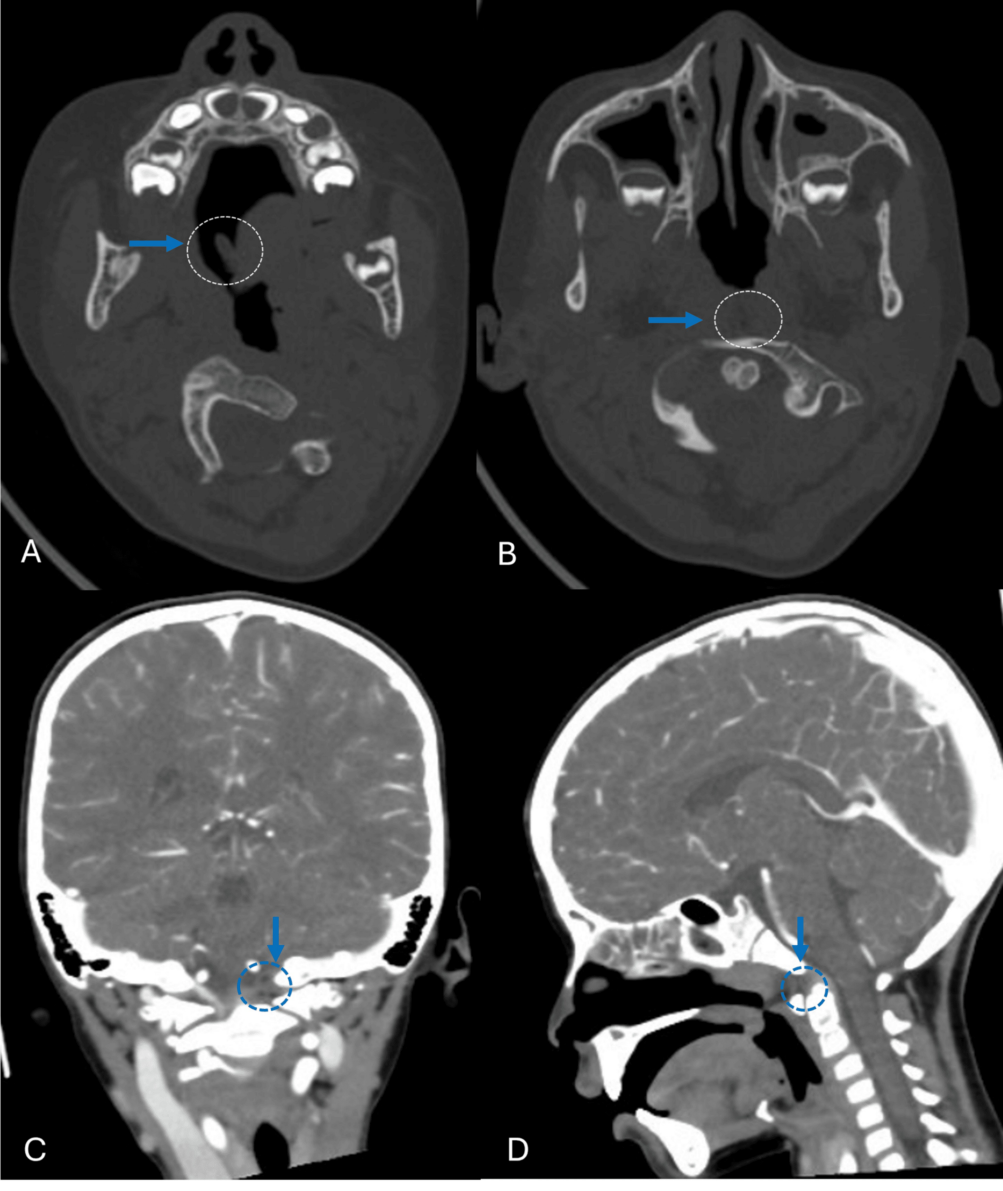
AngioCT cranial scan. (A, B) Axial bone window view, blue arrow showing the foreign object path. (C, D) Coronal and sagittal soft tissue window view, dotted circle showing the foreign body in close proximity to the upper portion of the odontoid process and reaching the foramen magnum, it does not enter the brain.

**Figure 3:**
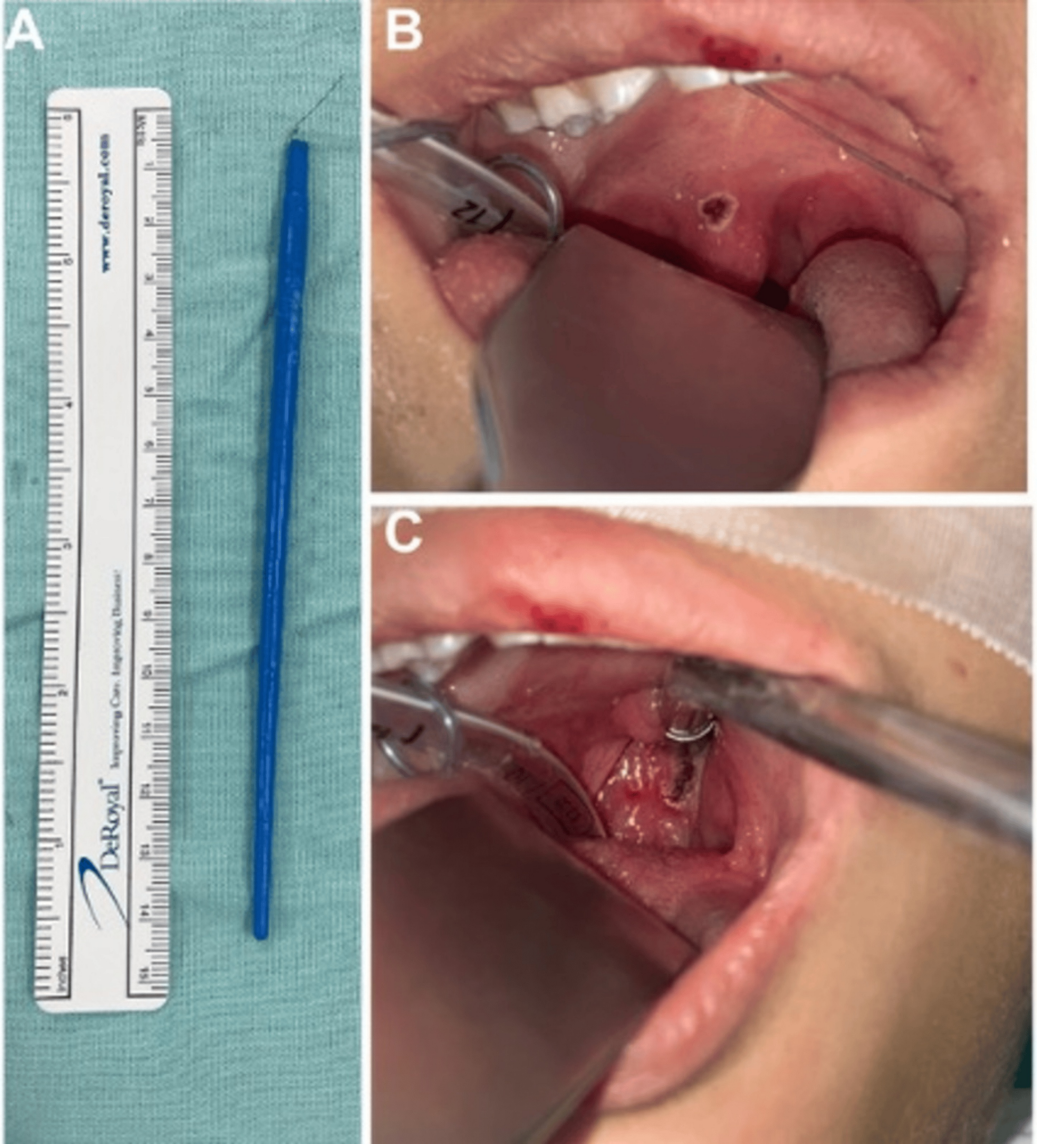
(A) Foreign object removed. (B) Nasopharynx injury after removing the object. (C) Soft palate injury after removing the object.

Computed tomography angiography of the head revealed the foreign body as a hypodense structure through the soft palate at the base of the uvula. The object followed a posterior path from right to left, crossing the posterior wall of the nasopharynx, passing between the base of the skull and the anterior arch of the atlas, in close relationship with the upper portion of the odontoid process. It reached the foramen magnum, displacing the meninges and the left vertebral artery in its V4 segment without causing injury. The meninges were observed intact. Additional findings included maxillary and ethmoidal sinusitis (Figures 2A-2D). There was no evidence of involvement of other important adjacent structures, such as the brain or the optic nerves. Due to the clinical and radiological findings, and the absence of intracranial structural injuries, the neurosurgery department recommended that management continue under the otorhinolaryngology department. An exploration in the operating room under balanced general anesthesia was performed. Under direct vision, careful traction with Kelly forceps was applied on the same axis as the object’s entry, achieving extraction with minimal bleeding from the nasopharynx and soft palate, which was controlled with cautery (Figures 3A-3C).

**Figure 4:**
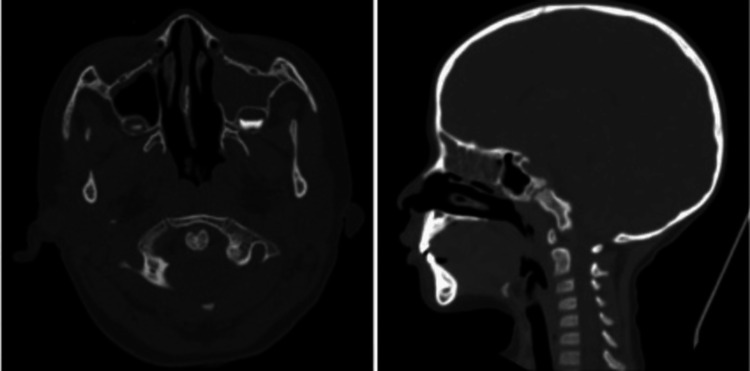
Simple CT scan after removing the foreign object showing no evidence of residual injury and air bubbles along the path of the foreign object.

**Figure 5:**
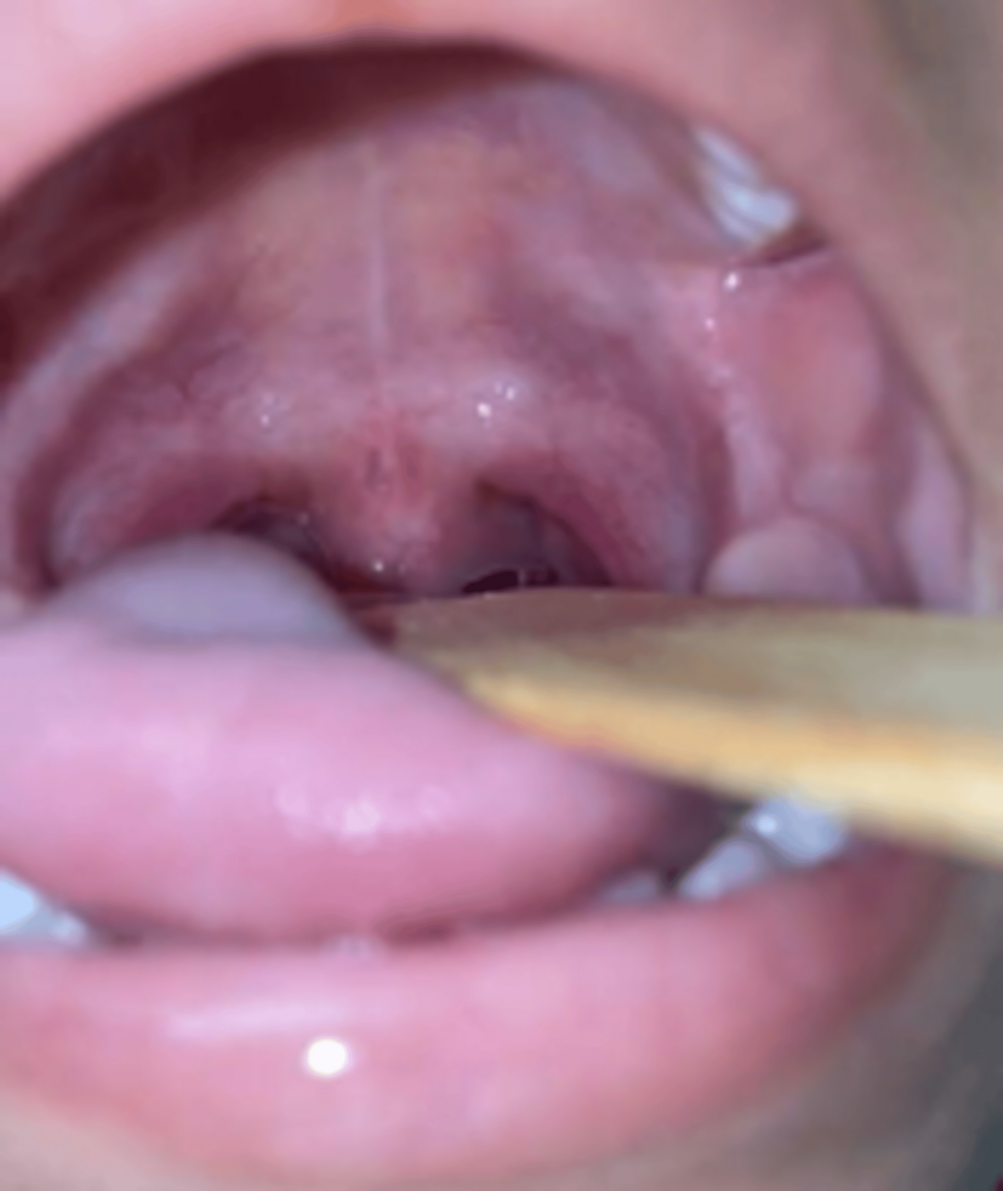
Healed soft palate in the first follow-up visit.

The patient was admitted to the post-operative observation unit for the first six hours. Subsequently, he was transferred to the general care ward due to stable hemodynamic and ventilatory status for control and surveillance, receiving ceftriaxone 500 mg IV every 24 hours and metronidazole 200 mg IV every 12 hours. During hospitalization, a feeding plan was implemented that included a liquid diet on the first day, which progressed to soft foods on the second and third days and ultimately reintroduced to a normal diet due to the patient's adequate tolerance. The postoperative CT scan showed no evidence of a foreign body and no damage to soft and bone tissues (Figure 4). The child was discharged on the third day of hospitalization due to satisfactory clinical progress. The first follow-up was conducted seven days after discharge, during which the patient was asymptomatic, and adequate healing of the wound on the soft palate was observed (Figure 5). In subsequent consultations, the patient remained asymptomatic after one month.

## Discussion

The patient presented a penetrating injury from the oral cavity to the base of the skull. Transoral penetrating injuries and impalement injuries of the soft palate and oropharynx are relatively frequent in the pediatric population. While these injuries have the potential for serious complications, such as vascular or central nervous system involvement, may include carotid trauma or thrombosis, impalement site infection, intracranial injuries, meningitis, and vertebral fractures, these complications are rare [8-11].

The mechanisms that can lead to these injuries include falling onto an object held in the mouth, exerting direct force on an object inside the mouth, or falling or running toward an object with an open mouth [12]. A comprehensive medical history is essential to understand the sequence of events, the nature of the foreign object, and any other contributing factors [2]. Initially, upon the patient's arrival in the emergency room, stabilization is performed in accordance with the Advanced Trauma Life Support (ATLS) Protocol. This is followed by a detailed cranial examination and the prompt use of diagnostic aids, including simple x-rays, computed tomography with angiographic analysis, and magnetic resonance imaging in selected cases [13,14]. The empirical administration of antibiotics is recommended, alongside the prompt removal of the foreign body, to prevent major complications such as neurological injury or infection [10-13]. Oral mucosal lacerations usually heal spontaneously. Repair is considered based on clinical criteria or in cases of large or open wounds [11].

In-hospital observation of the patient for no less than 48 to 60 hours following the trauma is essential, as this period is considered the interval of lucidity between the trauma and the appearance of neurological symptoms [15,16]. Multiple factors contribute to the prognosis, with the extent of the lesion being the most significant. Nevertheless, mortality is low if the brainstem or main vessels are not involved [17-19]. Outpatient follow-up of the patient is essential to monitor for any neurological changes. Caregivers should be vigilant for emergency symptoms such as sudden headaches, confusion, seizures, weakness or numbness in the limbs, difficulty speaking, or changes in vision, as these may indicate complications requiring immediate medical attention [2,10,20].

## Conclusions

This case underscores the rarity of transoral penetrating injuries in the pediatric population and contributes valuable information to the medical literature. Due to the potential risk of serious complications, such injuries require a multidisciplinary approach that includes obtaining an appropriate medical history and conducting tailored radiological examinations for each patient, along with prompt evaluation in the operating room. This highlights the necessity of thorough assessment and intervention to minimize risks and improve patient outcomes.
